# First Description of a Cluster of Acute/Subacute Paracoccidioidomycosis Cases and Its Association with a Climatic Anomaly

**DOI:** 10.1371/journal.pntd.0000643

**Published:** 2010-03-30

**Authors:** Ligia Vizeu Barrozo, Gil Benard, Maria Elisa Siqueira Silva, Eduardo Bagagli, Silvio Alencar Marques, Rinaldo Poncio Mendes

**Affiliations:** 1 Department of Geography, School of Phylosophy, Literature and Human Sciences, University of São Paulo, São Paulo, São Paulo State, Brazil; 2 Laboratory of Medical Investigation in Dermatology and Immunodeficiencies (LIM-56), Clinics Hospital of the Medical School, and Laboratory of Medical Mycology (LIM-53), Tropical Medicine Institute, University of São Paulo, São Paulo, São Paulo State, Brazil; 3 Department of Microbiology and Immunology, Institute of Biosciences of Botucatu, São Paulo State University, Botucatu, São Paulo State, Brazil; 4 Department of Dermatology and Radiotherapy, Botucatu Medical School, São Paulo State University, Botucatu, São Paulo State, Brazil; 5 Department of Tropical Diseases, Botucatu Medical School, São Paulo State University, Botucatu, São Paulo State, Brazil; Oswaldo Cruz Foundation, Brazil

## Abstract

**Background:**

Identifying clusters of acute paracoccidioidomycosis cases could potentially help in identifying the environmental factors that influence the incidence of this mycosis. However, unlike other endemic mycoses, there are no published reports of clusters of paracoccidioidomycosis.

**Methodology/Principal Findings:**

A retrospective cluster detection test was applied to verify if an excess of acute form (AF) paracoccidioidomycosis cases in time and/or space occurred in Botucatu, an endemic area in São Paulo State. The scan-test SaTScan v7.0.3 was set to find clusters for the maximum temporal period of 1 year. The temporal test indicated a significant cluster in 1985 (P<0.005). This cluster comprised 10 cases, although 2.19 were expected for this year in this area. Age and clinical presentation of these cases were typical of AF paracccidioidomycosis. The space-time test confirmed the temporal cluster in 1985 and showed the localities where the risk was higher in that year. The cluster suggests that some particularities took place in the antecedent years in those localities. Analysis of climate variables showed that soil water storage was atypically high in 1982/83 (∼2.11/2.5 SD above mean), and the absolute air humidity in 1984, the year preceding the cluster, was much higher than normal (∼1.6 SD above mean), conditions that may have favored, respectively, antecedent fungal growth in the soil and conidia liberation in 1984, the probable year of exposure. These climatic anomalies in this area was due to the 1982/83 El Niño event, the strongest in the last 50 years.

**Conclusions/Significance:**

We describe the first cluster of AF paracoccidioidomycosis, which was potentially linked to a climatic anomaly caused by the 1982/83 El Niño Southern Oscillation. This finding is important because it may help to clarify the conditions that favor *Paracoccidioides brasiliensis* survival and growth in the environment and that enhance human exposure, thus allowing the development of preventive measures.

## Author Summary

Paracoccocidioidomycosis is acquired through inhalation of spores released from filamentous forms of *Paracoccidioides brasiliensis* presumably present in the soil of endemic areas. However, successful isolation of the fungus from these areas has been rare, making it difficult to determine the fungus's ecological niche and the environmental factors that might influence the rate of infection and disease. Identifying clusters of acute paracoccidioidomycosis cases could potentially help at identifying these factors. However, there are no published reports of paracoccidioidomycosis outbreaks. We describe an undetected cluster of acute paracoccidioidomycosis cases diagnosed in 1985 in São Paulo State, Brazil, and investigate its association with climate. We observed that soil water storage was atypically high in 1982/83, and that the absolute air humidity in 1984 was much higher than normal, which may have favored, respectively, antecedent fungal growth in soil and conidia liberation in 1984, the probable year of exposure. This atypically high soil water storage was due to the strongest El Niño event in the area in the last 50 years, which also caused atypically high precipitation in that area. Better knowledge of the conditions that favor the fungus's survival and growth in the environment and that enhance human exposure may facilitate the development of preventive measures.

## Introduction

Paracoccidioidomycosis (PCM) is the most important endemic deep mycosis in Latin America [1]. Infection is acquired through inhalation of airborne propagules released by the filamentous forms of the fungus *P. brasiliensis* present in the soil of endemic areas. Although it has been estimated that, by 1994, over 10 000 cases had already occurred, its actual incidence and prevalence are not well known because it is not a notifiable disease in most countries where it is endemic, and because it is not equally distributed even in endemic areas [1]. Most frequently, individuals living in rural areas who have been exposed do not develop the disease. Studies in Colombia estimate that 9% of the population had had contact with the fungus while the incidence of the disease fluctuated between 0.05–0.22/100 000 inhabitants [2]. Clinically, the most common presentation is the chronic form [3]: the patients, mostly adult men, typically have involvement of the respiratory tract, from lungs to oral cavity, many years, sometimes decades, after the initial exposure. Rarely, the mycosis develops soon after exposure, the acute/subacute form (AF), causing a severe, disseminated disease that affects the reticuloendothelial system. In this case, both genders can be affected and the patients are younger.

Differently from most endemic mycoses (e.g., blastomycosis, coccidioidomycosis, and histoplasmosis), there are no published reports on outbreaks of PCM. In a previous work we described the influence of environmental factors on the incidence of the AF of this disease in an endemic area for a 31-years period [4]. Here, we identify a cluster of AF paracoccidioidomycosis cases diagnosed in this area at the interval of one year and the climatic anomalies that might be associated with this occurrence.

## Methods

This study was approved by the Committee for Ethical Research of the Medical School of São Paulo State University.

This is a retrospective study of the incidence of AF cases observed in the endemic area of Botucatu, São Paulo State, Brazil, from 1966 to 2006. This study area comprises 44 municipalities located in the central-western region of São Paulo State, Brazil, where the altitudes vary from 450 to 1008 m above sea level and the mean air temperatures varies from 19.3 to 22.5°C. Predominant land use types are pasture, croplands, and *Pinus* and *Eucalyptus* plantations. Remnant natural vegetations are savanna-like and mesophytic and riparian forests. Mean annual precipitation ranges from 1272 to 1589 mm.

This area is served by a single public hospital, the University Hospital (UH) of UNESP, to which all patients suspected of having PCM are necessarily referred. From 1966–2006, 825 cases with confirmed diagnosis of PCM were registered. Among these, 484 resided within the study area and 96 (19.8%) presented the AF PCM. Patients included in this study had the diagnosis of PCM confirmed by the identification of the agent in clinical specimens and were classified as having the AF of the disease based on published criteria [3]. From those 96 patients, 67% were up to 24 years old and 66% were males. We then performed a retrospective cluster detection test to verify if an excess of cases in time and/or space occurred. Cases were assigned to a particular year based on the date of diagnosis. Cases were geocoded by municipalities based on the centroid of the polygonal geometry of each municipality. Calculations were performed with the software SaTScan v7.0.3 [5]. Cases were assumed to be Poisson distributed, adjusted for age and gender with constant risk over space and time under the null hypothesis. Cluster analysis results include temporal and space-time clusters with no geographic overlap of clusters allowed and a maximum allowable cluster size of 50% of the population. The scan test was set to find clusters for the maximum temporal period of 1 year. Significance was evaluated with Monte Carlo simulation with 9999 replications where the null hypothesis of no clusters was rejected at an α level of 0.05.

## Results

Mean cases by year was 2.37 (SD: 1.74) and mean incidence was 0.4 annual cases/100 000 inhabitants. The temporal test indicated a significant cluster in 1985 (*P*<0.005). This cluster comprised 10 cases when 2.19 were expected for this year, resulting in a relative risk of 4.98 (Table 1). Age range was 5–27 years old, 6 were females and 4 males, and the manifestations preceded the diagnosis by less than 1 month to six months. Clinical presentation was typical of the AF PCM. Involvement of the reticuloendothelial system was the hallmark: 8 had lymphadenopathies, either superficial or deep (abdominal/mediastinal), and 6 had either hepatomegaly or splenomegaly. Other less frequent involvements were skin (3 patients), bone (1 patient) and mucosal lesions (1 patient). None had pulmonary involvement on physical examination and chest X-rays. With regard to the year of infection, it is estimated that a period of an average of 11 months exists between the moment of exposure and date of diagnosis [6]. This period takes into account the incubation time of the mycosis and the time that the patient with symptoms takes to search a medical service and a diagnostic test is provided [4]. Thus, the probable year of infection for this cluster was 1984.

**Table 1:** Clusters of Acute/subacute PCM, in the Region of Botucatu, Brazil, 1966–2006 (n = 96).

Cluster type	Municipalities^a^	Period	Cases	Expected	RR^b^	*p-*value
Temporal	All	1985	10	2.19	**4.98**	**0.0014**
Space-time	Angatuba, Guarei, Paranapanema, TorredePedra, Itatinga, Bofete, Pardinho, Porangaba, Avare, Itai, Botucatu, Arandu, Pratania, Taquarituba	1985	8	0.89	**9.77**	**0.0081**

a These municipalities are depicted in Figure 1.

b RR, relative risk.

The space-time test confirms the temporal cluster in 1985 and shows the location where the risk was higher in that year, including 14 municipalities (Table 1 and Figure 1). The space-time cluster included 8 cases when 0.89 was expected for that location, with a relative risk of 9.77. Some environmental variables that were significant for modeling the entire series of data were analyzed: soil water storage and absolute air humidity, as previously determined [4]. Soil water storage is the amount of water that is kept in the soil after computing the annual gains of water as precipitation and loses by evapotranspiration. Sequential water balances were calculated based on field data for daily precipitation, collected through 37 rain gauges distributed in the study area and calculated using the Thornwaite and Mather water balance model [7].

**Figure 1:**
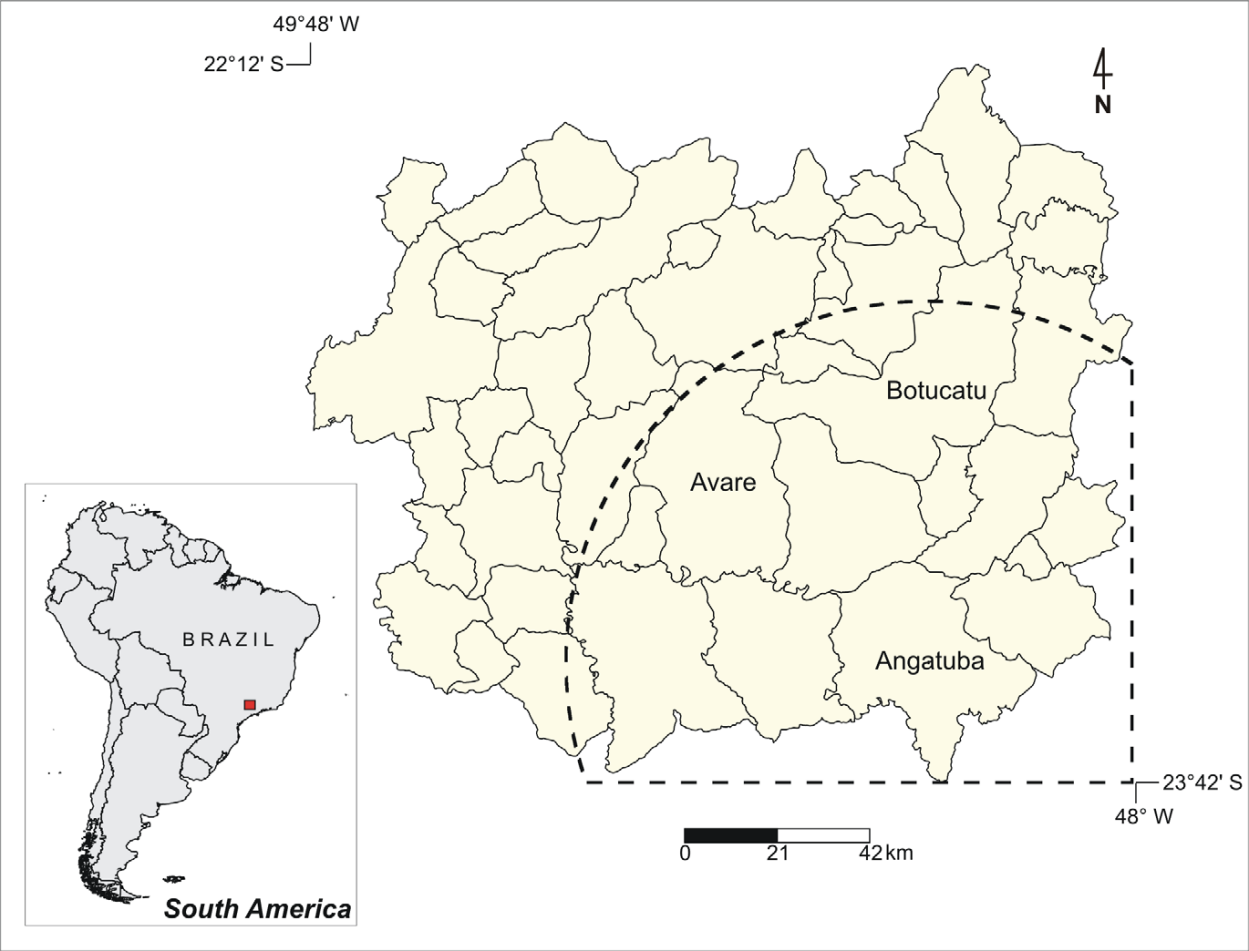
Municipalities in the study area overlaid by the space-time cluster localization (dashed line).

The cluster suggests that some particularities took place in the antecedent years in those localities. Diagnostic or reporting methods of PCM could explain variation in incidence. However, practices of the UH were reviewed and it was not detected any modification in the diagnostic methods or reporting methodology. Type of land use in this same area and period was investigated previously and no abrupt change was detected that could also be associated with this cluster [8]. Instead, we observed a significant close relationship of climatic factors with incidence of AF in the entire series of data [4]. Previous modeling study showed that the most significant required climatic factors are increase of soil water storage 2 years before the probable year of infection combined with higher absolute air humidity in the year of infection. Calculated soil water storage was atypically high in the years of 1982/83 (∼2.11 and 2.5 standard deviations above mean), especially in the municipalities where the cluster occurred, and the absolute air humidity in 1984, the year preceding the cluster, was much higher than normal (∼1.6 standard deviations above mean), conditions that, may have favored, respectively, antecedent fungal growth in the soil and conidia liberation in 1984, the probable year of exposure.

## Discussion

Environment variables and/or climate are known to influence endemic mycoses incidence [9]–[11]. For example, a recent epidemic of coccidioidomycosis in Arizona was associated with changes in local climatic and environmental variables [12]. However, a relationship between a global climatic anomaly and the abrupt change in a mycosis incidence has not yet been found [13]. We describe here the first well-documented cluster of cases of AF PCM, which potentially bears a relationship with a climatic anomaly, namely the 1982-83 El Niño event.

The patients in this cluster presented the range of clinical manifestations expected for the AF disease, with no deaths recorded during the first year of follow-up. The pattern of organ involvement reproduces closely that one described in a recent compilation of AF cases [14]. All cases responded well to the medications during the first year of treatment. Thus, the cases of this cluster apparently did not differ grossly from what is described for the AF of the disease [14], and do not suggest the participation of more pathogenic isolates. Moreover, no clustering of chronic form cases was detected at this year probably because this presentation has long and variable latency periods.

The reasons why clusters of AF PCM, differently from blastomycosis or coccidioidomycosis, have never been documented are not fully known. To date, few variations in incidence have been observed [2]. Nonetheless, an increase in the number of chronic form cases occurred among Amazonian Suruí Indians within 1983–1986, after abrupt change in land use in a previously unexplored forest area [15]. The environmental changes related to these cases consisted in clear-cutting the forest trees for the development of coffee farming that started a few years before [15]. Interestingly, an atypical increase in AF cases was reported also in the eastern Brazilian Amazon in 1988/89, with most patients residing in Imperatriz, State of Maranhão, but was no further investigated [16].

We hypothesize that this cluster was linked to a climatic anomaly caused by the El Niño Southern Oscillation (ENSO) phase in 1982-83. ENSO is a widespread oscillation of sea surface temperature between the east and west regions over the Equatorial Pacific. This oscillation presents interannual variability and is linked to the atmosphere through sea-level pressure and wind anomalies. In Brazil, the ENSO behavior explains a large part of the interannual rainfall variability near the equatorial regions and in the southern [17]. The atypically high soil water storage in 1982/83 occurred due to the strongest El Niño event in the last 50 years, which also caused precipitations higher than two standard deviations above mean in our study area [18]. El Niño has been implicated in the dynamics of transmission of several infectious diseases [19]. However, in PCM, beyond the difficulties in identifying clusters of this disease, the link with other El Niño events is not always evident because no two ENSO events are exactly alike, differing in intensity, timing and spatial organization, and the extra-tropical climatological and ecological responses similarly vary from event to event [18].

A possible limitation of our study is its retrospective nature, which did not allow us to determine other potential commonalities among the patients that could be related to exposure to *P. brasiliensis*, such as living in proximity to river banks, specific soil related activities, hunting of armadillos and others. [1] Another limitation was the lack of identification of other significant clusters in this area which could eventually strengthen the link with climate anomalies and permit to establish a causal connection.

We hypothesize that clusters of PCM may occur but remain undetected mainly due to logistical reasons. First, PCM still is not a notifiable disease in most countries where it is endemic. Second, differently from our endemic region, the registry of patients with the AF of the disease occurring at an endemic area tends to be dispersed because frequently these patients find their diagnosis and treatment at different distant medical centers. PCM still poses several challenges especially regarding the identification of *P. brasiliensis* ecological niche. Identifying clusters of PCM in different geographical areas is important because it may help to clarify the conditions that favor *P. brasiliensis* survival and growth in the environment and enhance human exposure, thus allowing the development of preventive measures.
